# 

**Published:** 1900-07-07

**Authors:** 


					??????  _ - _ _ -.Tiyi/"1"' ' =- t/Uiil 1UW?
KUllUn^U UA lAA^AlVOk pui IIJT ?
CADBURY'S rf* H ^ CADBURY'S
r*r\r+r\ a \eV Jik /Mi Je5L^ COCOA
COCOA )1k ^ ^ ML? COCOA
ABSOLUTELY PURE. NO DRUGS OR ALKALIES USED.
Lascow Western Infirmar
ascan Western InfYrman?
No. 719. Vol. XXTOI. paSSta Satubdat,
July 7, 1900.
? ^^fJDWOLKAui NOR IY/CH
rSnbsoription Terms,~|
l_ see p. 2S4. J
PRICE TWOPENCE.

				

